# Isolated Pulmonary Valve Infective Endocarditis With Persistent Staphylococcus aureus Bacteremia and Rapid Clearance With Ertapenem Plus Cefazolin

**DOI:** 10.7759/cureus.77335

**Published:** 2025-01-12

**Authors:** Paulo Fernandes, João Maia Oliveira, Ana Rita Rocha, Sara Carvalho, José Vaz

**Affiliations:** 1 Intensive Care Unit, Hospital José Joaquim Fernandes, Beja, PRT; 2 Internal Medicine Department, Hospital José Joaquim Fernandes, Beja, PRT

**Keywords:** ertapenem plus cefazolin, infective endocarditis, methicillin-sensitive staphylococcus aureus, necrotizing pneumonia, persistent bacteremia, pulmonary valve endocarditis

## Abstract

Infective endocarditis is an infection of the heart’s native or prosthetic valves, often caused by bacteria such as *Staphylococcus aureus*. Although infective endocarditis most commonly affects the left heart, cases of right-sided infective endocarditis, involving structures like the tricuspid or pulmonary valves, are also noted. Isolated native pulmonary valve infective endocarditis is exceptionally rare. After suspicion, the diagnosis relies on clinical symptoms and signs, imaging, and microbiological evidence.

We report an unusual case of isolated pulmonary valve infective endocarditis in a previously healthy 59-year-old man without typical risk factors. He presented with unspecific symptoms such as fever, chills, dizziness, and left shoulder pain. Radiologically, the patient presented small ground-glass opacities in both lungs that aggravated during the first days after hospital admission with multifocal consolidation areas, and later developing bilateral necrotizing pneumonia. Despite adequate antibiotic treatment, the patient developed septic shock and persistent *Staphylococcus aureus* bacteremia. Given the persistence of such microorganisms in the bloodstream, despite the initial absence of endocardial involvement on transthoracic echocardiography, transesophageal echocardiography was done and revealed a large vegetation on the pulmonary valve with valvular regurgitation. According to Duke's criteria for infective endocarditis, a definite diagnosis was made, once both major clinical criteria were present, namely, typical microorganisms consistent with infective endocarditis from two separate blood cultures and evidence of endocardial involvement. Given the refractory bacteremia, an unusual combination of antibiotic therapy, including ertapenem and cefazolin, was introduced, leading to rapid clearance of bacteremia. This salvage antibiotic regimen was chosen due to the synergy of carbapenem with cefazolin and their potential improved bactericidal activity within biofilms. The patient subsequently required surgical intervention with bioprosthetic pulmonary valve replacement and ultimately achieved near-full recovery after a prolonged hospital stay.

This case illustrates the diagnostic and therapeutic challenges of rare right-sided infective endocarditis since the patient presented with non-specific symptoms, without typical risk factors for right-sided infective endocarditis and the initial transthoracic echocardiography showed no valvular vegetations. Furthermore, the persistence of bacteremia despite adequate antibiotic therapy was a clinical challenge and this case highlights the potential efficacy of ertapenem plus cefazolin in treating persistent *Staphylococcus aureus* infections. It underscores the importance of individualized management in severe cases and the need for ongoing research to optimize treatment strategies for persistent infections.

## Introduction

Infective endocarditis (IE) can affect native or prosthetic heart valves, intracardiac devices, and, less frequently, nonfunctional embryonic remnants in the right atrium. It occurs when these structures become infected by bacteria or, more rarely, fungal organisms [1]. Right-sided IE represents only 5-10% of all IE cases [2], though the overall incidence has been rising recently [3] due to the increased survival of patients with risk factors, which include implanted cardiac devices, congenital valve disorders, diabetes, immunosuppressive treatments, and end-stage renal disease requiring dialysis [4]. Additional risk factors include intravenous drug use, central venous catheters, and prosthetic heart valves [4].

In right-sided IE, the tricuspid valve is more frequently involved than the pulmonary valve. Isolated pulmonary valve IE is an extremely rare condition that accounts for 1.5-2% of all reported cases of IE ​[5]. Besides its rarity, vegetation may be difficult to identify on the pulmonary valve, even by transesophageal echocardiography.

The diagnosis of IE is based on clinical, echocardiographic, and microbiological findings. The modified Duke major criteria are the mainstay of IE diagnosis and consist of two separate positive blood cultures of typical microorganisms consistent with IE (*Viridans streptococci*, *Streptococcus gallolyticus*, HACEK (*Haemophilus* species, *Aggregatibacter* species, *Cardiobacterium hominis*, *Eikenella corrodens*, and *Kingella* species) group, or *Staphylococcus aureus*) and the presence of valvular vegetations detected by echocardiography ​[6].

One of the most common pathogens causing bacteremia is *Staphylococcus aureus,* which is also the leading cause of IE. Persistent methicillin-susceptible *Staphylococcus aureus* (MSSA) bacteremia (for at least three days) is a predictor of mortality *per se*, and increases the likelihood of metastatic infections ​[7,8], aggravating the clinical scenario. Several case series have shown that the combination of ertapenem and cefazolin effectively cleared refractory MSSA bacteremia, due to the synergy of carbapenem with cefazolin and their potential improved bactericidal activity within biofilms ​[9,10].

We present a challenging case of a 59-year-old male with isolated native pulmonary valve IE, without risk factors, in which a “second-line” antibiotic regimen had to be started due to persistent MSSA isolation in blood cultures despite adequate antibiotic therapy, with rapid resolution of bacteremia.

## Case presentation

**Figure 1:**
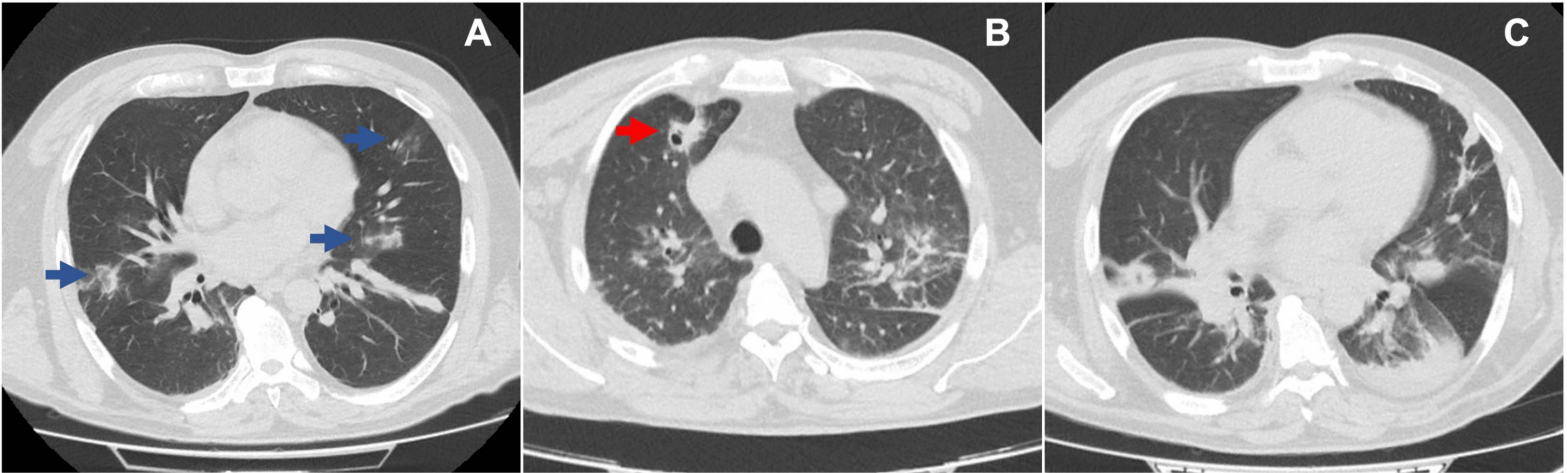
Computed tomographic pulmonary images. (A) On hospital admission, with small ground glass areas bilaterally (blue arrows). (B and C) On day three after hospital admission, showing bilateral areas of consolidation and cavitation in the right upper lobe (red arrow).

A 59-year-old immunocompetent male, who was a rural worker, without relevant past medical history, presented to the emergency department with a five-day history of fever, chills, dizziness, left shoulder pain, and anorexia. He reported having a small pre-tibial skin wound that resolved spontaneously about three weeks before. On clinical examination, he was normotensive, tachycardic with 121 beats per minute (sinus rhythm on electrocardiography), with a body temperature of 37.5°C and oxygen saturation (SpO2) of 94% on room air. He was asthenic, with global weakness, and the left shoulder was painful on palpation with passive mobilization. The rest of the physical examination was unremarkable. A cerebral computed tomography (CT) scan showed no alterations, but a thoracic CT scan revealed mild spots with ground glass patterns in the middle, lingula, and right lower lobes (Figure 1).

Although not clinically suggestive, given the presence of shoulder pain, fever, and elevated inflammatory markers (Table 1), septic arthritis of shoulder joints was excluded by a CT scan, showing the absence of effusion in shoulder joints. Laboratory investigations on admission are presented in Table 1. The patient's renal function was impaired since the baseline creatinine level was 0.8 mg/dL before admission. The inflammatory markers were elevated (total leucocyte count and serum C-reactive protein) and he had moderate thrombocytopenia, interpreted in the context of infection. Blood and urine cultures were collected and the patient was admitted to a medical ward for further investigation. Amoxicillin-clavulanate was started empirically.

**Table 1:** Laboratory investigations at hospital admission.

Parameters	Results	Reference range
Hemoglobin	15.7 g/dL	13.2-16.6 g/dL
Total leucocyte count	12,500 /uL	3,800-8,760/uL
Platelet count	74,000 /uL	173,000-360,000/uL
Serum urea	62 mg/dL	15-38 mg/dL
Serum creatinine	1.67 mg/dL	0.7-1.3 mg/dL
C-reactive protein (CRP)	25.10 mg/dL	<0.3 mg/dL
N-terminal pro-B-type natriuretic peptide (NT-proBNP)	1,518 pg/mL	<125 pg/mL

During the first five days after admission, the patient's condition deteriorated as he developed respiratory failure and altered mental status with confusion. Radiologically, there was an increase in the parenchymal opacities, with areas of small cavitations, which eventually evolved into bilateral necrotizing pneumonia. The admission blood cultures isolated MSSA. The same microorganism was isolated in sputum culture.

**Figure 2:**
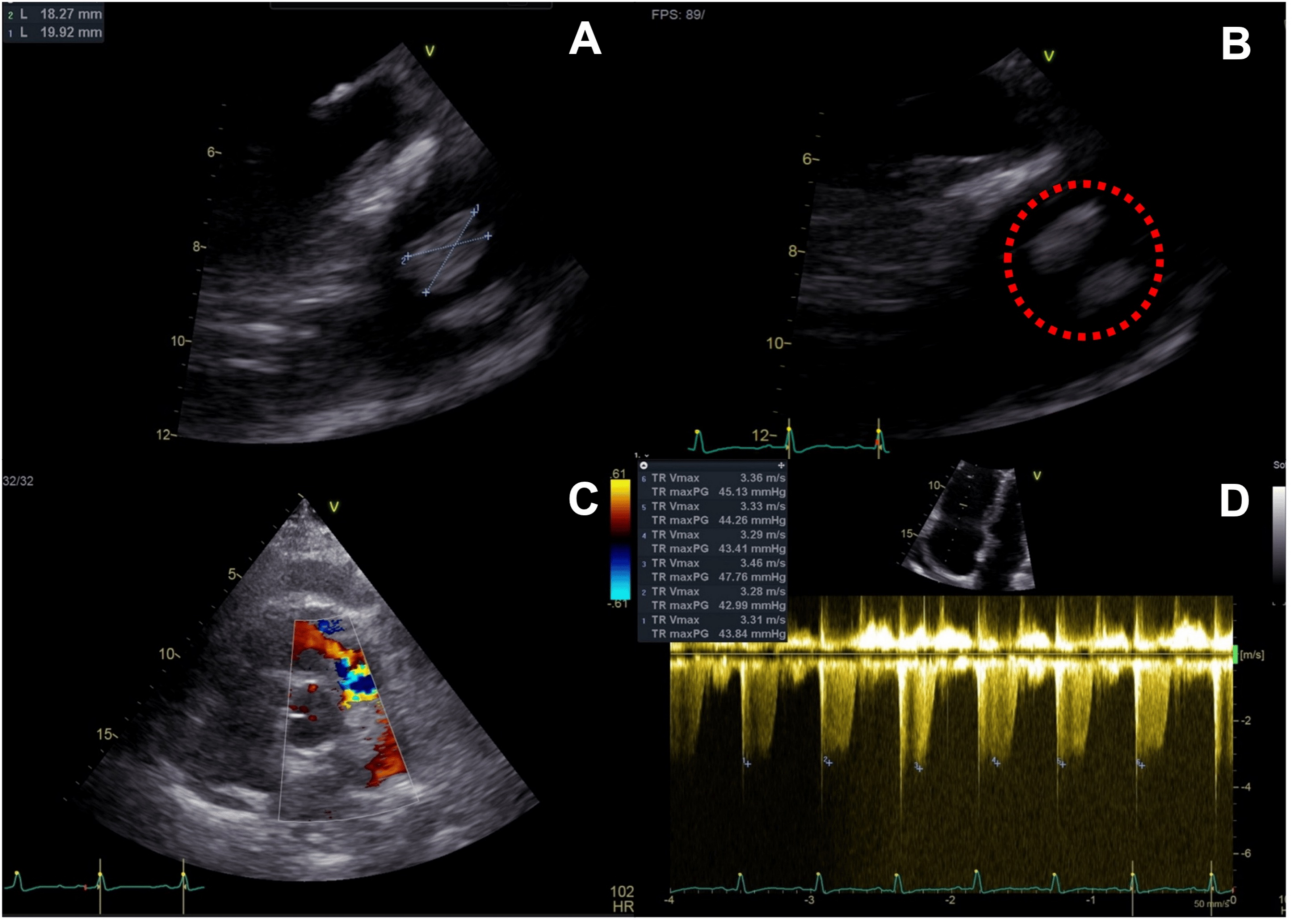
Pulmonary valve vegetation on echocardiography. (A and B) Transesophageal echocardiography revealing pulmonary valve vegetation (red circle) with approximately 20 mm diameter. (C) Transthoracic echocardiography documenting significant pulmonary valve regurgitation with color Doppler. (D) Right ventricle dilation with peak systolic transtricuspid pressure gradient of 45 mmHg.

He was admitted to the intensive care unit (ICU) due to respiratory failure. Given the refractory MSSA bacteremia, the antibiotic was changed to linezolid. Although the *Staphylococcus aureus* was oxacillin-sensitive, the choice of linezolid was based on the global severity of the patient, the known pulmonary infection, and the persistent deterioration under amoxicillin-clavulanate therapy. On ICU admission, transthoracic echocardiography showed no evidence of valvular vegetation. However, the persistence of MSSA bacteremia and multifocal pulmonary opacities led us to suspect a right-sided IE and the ICU team requested a transesophageal echocardiography (TEE). TEE identified an isolated pulmonary valve vegetation measuring 20 x 18 mm (Figure 2) causing severe pulmonary valve insufficiency and right ventricle dilation. The other valves were intact. Clinically, these findings are traduced in progressive right-heart failure.

The diagnosis of isolated native pulmonary valve endocarditis complicated with severe pulmonary valve regurgitation and septic pulmonary embolism was made. The respiratory failure was successfully managed with non-invasive ventilation and a high-flow nasal cannula, without the need for invasive mechanical ventilation. In the first days of the ICU stay, he developed septic shock and started vasopressor support with norepinephrine at a maximum infusion rate of 0.8 mcg/kg/min. Hydrocortisone 200 mg per day was added as an adjunctive therapy for septic shock. The fluid resuscitation was managed carefully and with frequent hemodynamic reassessment by echocardiography, given the right heart failure.

Blood cultures were repeated every two days and after seven days of linezolid, the patient persisted with MSSA bacteremia, so the antibiotic was switched to flucloxacillin. Despite this change, the patient developed multiple organ dysfunction.

**Figure 3:**
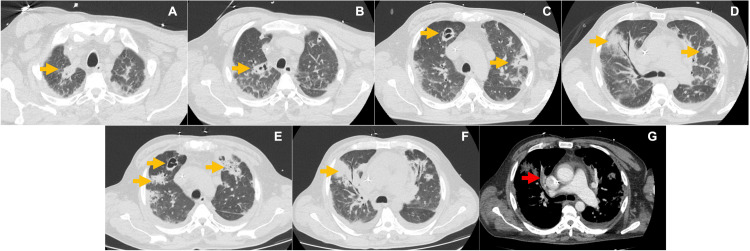
Temporal evolution of lung CT scans showing progression of pulmonary consolidations. (A-D) Day nine after admission. (E-G) Day 18 after admission. Yellow arrows indicate multiple septic consolidations, with some nodular cavitations and the red arrow indicates a filling defect of the middle lobar artery.

A subsequent chest CT angiography revealed an increase in the number of pulmonary parenchymal opacities and pulmonary embolism (Figure 3). Despite the etiology of the pulmonary embolism was septic dissemination from the pulmonary valve, concomitant thromboembolic embolism could not be excluded. The benefits and risks of anticoagulation were discussed, and therapeutic enoxaparin was initiated. As the bloodstream infection remained uncontrolled after 12 days of *in vitro* effective antibiotics, a combination of ertapenem plus cefazolin was initiated, with blood cultures returning negative after 48 hours. This salvage antibiotic regimen was chosen due to the synergy of carbapenem with cefazolin and their potential improved bactericidal activity within biofilms.

Considering the magnitude of valvular dysfunction and the pulmonary metastatic infection, the patient was referred to a cardiothoracic center. A total of 34 days after the first negative blood culture, the patient was submitted to a bioprosthetic pulmonary valve replacement. The microbiological culture of the native valve was negative. Pleuro-mediastinal hemorrhage complicated the immediate postoperative period, which led to an early re-sternotomy due to the magnitude of the hemorrhage, with effective hemostasis. During the following weeks, the patient developed a right pleural empyema due to *Escherichia coli*. The empyema was drained with a chest tube and treated with carbapenem. Twenty days later, the empyema persisted, and a right lower lobe decortication was performed.

After 127 days of admission, the patient was discharged home to a physical rehabilitation program, which allowed him to restore the pre-admission status performance, on ICU follow-up appointment.

**Figure 4:**
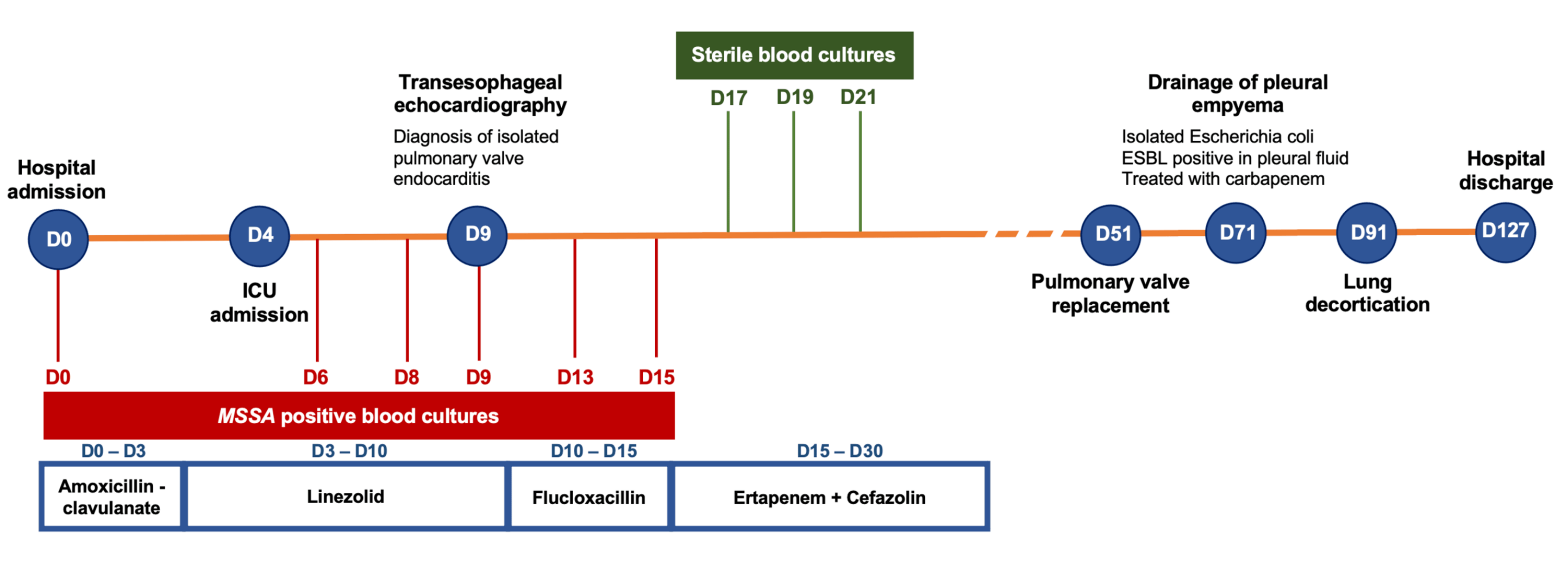
Timeline summarizing the main events during the hospital stay, the antibiotics administered during the first 30 days, and the blood cultures collected during this period. ESBL: extended-spectrum beta-lactamase; MSSA: methicillin-susceptible *Staphylococcus aureus*.

Figure 4 summarizes the main events during the hospital stay.

## Discussion

As mentioned before, right-sided IE is a rare phenomenon, and isolated pulmonary valve IE is even rarer. One possible explanation for this low incidence is the relatively low pressure gradient across the pulmonary valve, resulting in less shear stress compared to other valves. This reduces the likelihood of valvular damage, thereby offering some protection against IE. Additionally, valvular abnormalities in the pulmonary valve are infrequent, which further decreases its susceptibility to the development of vegetation ​[11].

The patient had no major risk factors for developing a right-sided IE, such as injection drug use, indwelling cardiac devices, or other intravascular devices, which reinforces the atypical nature of this case. In retrospect, given the microorganism isolated in blood cultures and the presence of right-sided IE, the probable source of bacteremia was the skin wound he had weeks before presentation to the emergency department since no other entry portals were identified. The initial ground-glass opacities found on CT scans, although non-specific, were revealed as a precursor for the multifocal consolidation areas with cavitations and necrotizing pneumonia.

The detection of *Staphylococcus aureus* bacteremia allowed early directed antimicrobial therapy, first with linezolid and then narrowing to flucloxacillin. However, despite targeted antibiotic therapy, the patient continued to deteriorate clinically and radiologically and the bacteremia persisted after 12 days of in vitro effective antibiotics. Although the first transthoracic echocardiography was not suggestive of valvular vegetations, the persistence of bacteremia and the presence of bilateral pneumonia led the ICU team to suspect a right heart IE. A TEE was then requested, as it has higher sensitivity for right-sided IE, especially on pulmonary valve vegetations.

The use of linezolid might have some limitations. Despite effective activity *in vitro* against MSSA, its use might be limited due to the bacteriostatic effect. Since the persistence of the microorganism isolated in blood cultures always presented the same resistance pattern, linezolid was changed to flucloxacillin. The major factors contributing to the persistent bacteremia were (1) the dimension of the vegetation, (2) the metastatic dissemination of the infection to the lungs, (3) the fact that the microorganism was a *Staphylococcus aureus*,* *which tends to persist in the bloodstream despite the presence of microbiologically appropriate antibiotics, and (4) the propensity of MSSA to form biofilm within native host tissues ​[12]. Furthermore, IE due to *Staphylococcus spp.* seems to be an independent predictor of worse in-hospital outcomes ​[11]. This led the ICU team to find an alternative antibiotic regimen.

The combination of carbapenems with piperacillin-tazobactam has been demonstrated to be effective against methicillin-resistant *Staphylococcus aureus* (MRSA) *in vitro* and *in vivo*, and some older* in vitro* studies have shown a synergistic effect between cephalosporins and carbapenems ​[9,10]. More recent studies have demonstrated the efficacy of clearance of MSSA bacteremia with the combination of cefazolin plus ertapenem. The rationale for selecting this combination in these studies was to provide therapy with two beta-lactam antibiotics with complementary penicillin-binding protein (PBP) proclivities, thus simultaneously targeting multiple steps in cell wall synthesis to provide enhanced bacterial killing. Specifically, carbapenems have an exceptional affinity to the essential PBP of *Staphylococcus aureus* (PBP1), which would complement the relative PBP2 proclivity of cefazolin ​[13,14]. Besides, this combination might enhance the antibiotic activity within biofilm [15], translating​ into a shorter duration of bacteremia in patients with MSSA endocarditis.

Given these findings, the team opted to use this approach to treat refractory MSSA bacteremia in a case where no alternative options were available except to wait for clearance. In fact, the bacteremia was cleared in less than 48 hours in this case. Clinical improvement was observed concurrently, with the resolution of fever.

## Conclusions

This case illustrates the complexities of diagnosing and managing pulmonary valve IE caused by MSSA in a previously healthy patient, from diagnostic confirmation to effective source control. The rapid progression from initial symptoms to septic shock highlights the aggressive nature of this infection, the need for early recognition, and the benefit of multidisciplinary involvement (intensive care, cardiology, infectious diseases, and cardiac surgery teams). Future research exploring antibiotic synergy in persistent MSSA infections and larger clinical studies evaluating dual beta-lactam therapy are warranted to explore effective strategies for recurrent or persistent infections. Additional approaches for earlier detection of isolated pulmonary valve IE are also needed.

Overall, this case enhances our understanding of right-sided IE through its rarity, diagnostic challenges, and therapeutic approach. It also expands the discussion surrounding the management of complicated bacterial infections and the importance of tailored interventions.
